# Ethambutol‐induced subacute cutaneous lupus erythematosus

**DOI:** 10.1002/kjm2.12571

**Published:** 2022-06-29

**Authors:** Yi‐Wei Huang, Wei‐Hsin Wu

**Affiliations:** ^1^ Department of Dermatology National Taiwan University Hospital Taipei Taiwan

A 61‐year‐old man with a diagnosis of cervical tuberculous lymphadenitis, presenting initially with a unilateral progressive neck mass, was administered first‐line quadruple therapy (isoniazid 300 mg/day, rifampin 600 mg/day, ethambutol 1200 mg/day, and pyrazinamide 1500 mg/day) for 2 months. Subsequently, triple antituberculous treatment (isoniazid, rifampin, and ethambutol) was administered for additional 3 months. He presented to the dermatologic clinic with mildly itchy rashes that had appeared over the preceding 2 weeks. He denied history of ultraviolet‐induced rash or arthralgia. On examination, several polycyclic violaceous plaques, 1–3 cm in diameter, arranged in a photodistributed pattern involving the upper chest, upper back, and upper extremities (Figure 1). Immunological tests revealed positivity for antinuclear antibodies (1:640, homogeneous pattern). A test for anti‐Ro/SSA antibodies was positive (>240 U/ml), whereas tests for anti‐La/SSB antibodies and anti‐double‐stranded DNA antibodies were negative. Histopathological examination showed basal vacuolar degeneration and pigment incontinence along with lichenoid infiltrate of lymphocytes in the upper dermis. Immunohistochemical staining revealed some CD123+ plasmacytoid dendritic cells in lymphocytic infiltration. Direct immunofluorescent studies were negative for the deposits of immunoglobulin G (IgG), IgA, IgM, and C3. Overall, the histopathological findings were consistent with subacute cutaneous lupus erythematosus (SCLE).

**FIGURE 1:**
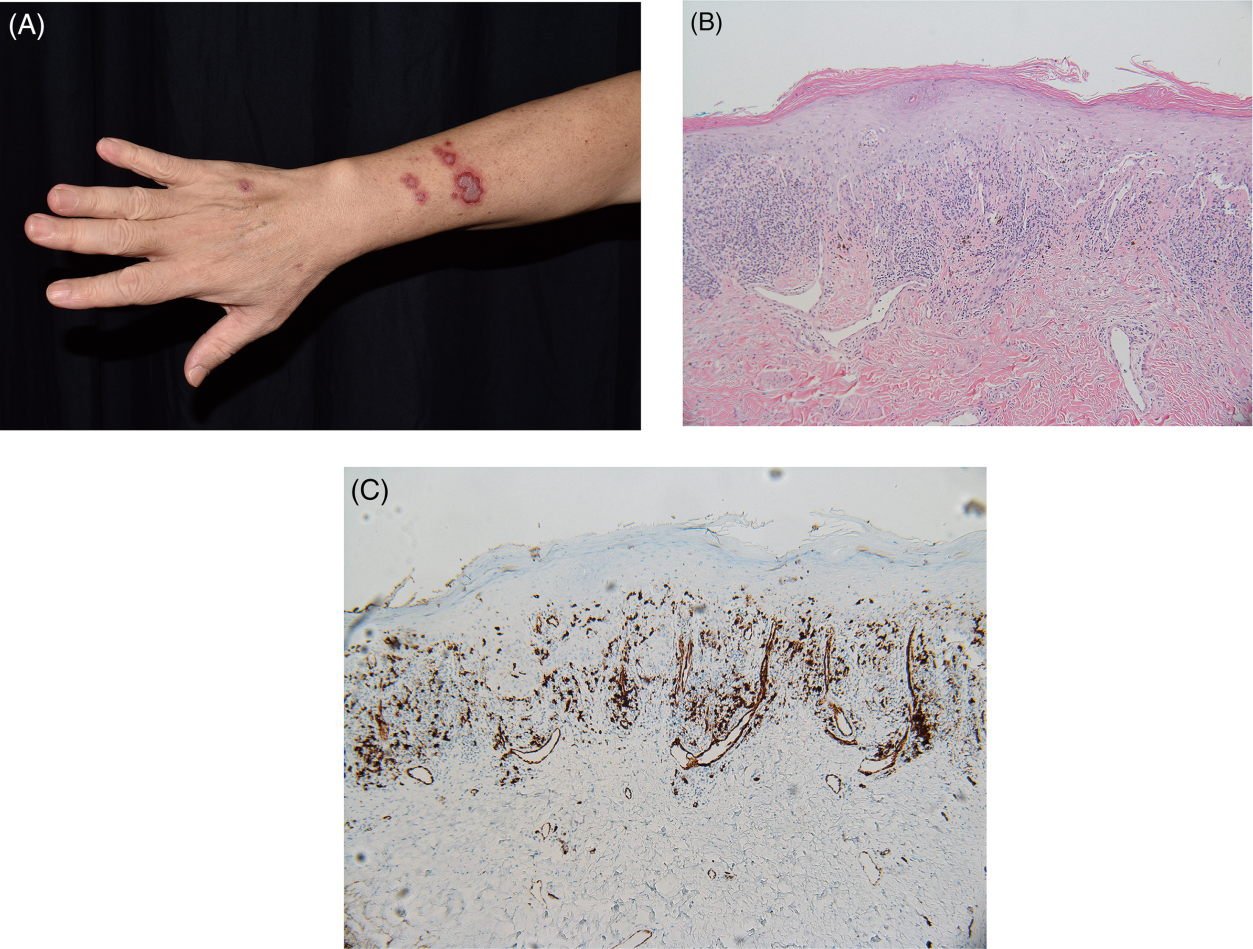
(A) A 61‐year‐old man presented with annular violaceous plaques on the forearm, demonstrating cutaneous eruptions of subacute cutaneous lupus erythematosus. (B) Lichenoid infiltration in the upper dermis and vacuolar degeneration of the basal keratinocytes. [hematoxylin and eosin] (C) Immunohistochemical staining shows CD123+ plasmacytoid dendritic cells in the lymphocytic infiltration.

His antituberculosis regimen was stopped, and oral hydroxychloroquine at 400 mg per day and topical clobetasol ointment were prescribed. Skin eruptions regressed in the subsequent 2 weeks. The patient was rechallenged with isoniazid and rifampin on the basis of the clinical judgment of an infectious disease specialist. The patient completed the remaining period of the 6‐month treatment course without the cutaneous manifestations recurring. The skin rash resolved completely, and the titers of antinuclear antibodies (1:640+) and anti‐Ro/SSA (>240 U/ml) antibodies were stationary at 13‐month follow‐up.

Drug‐induced SCLE (DI‐SCLE) is predominantly reported in middle‐aged women, and the mean incubation period is 27.9 weeks.^1^ Histopathologically, patients with DI‐SCLE show interface dermatitis and a lichenoid tissue reaction.^1^ Anti‐Ro/SSA is an autoantibody more specifically associated with DI‐SCLE, which is different from histone autoantibodies found in drug‐induced systemic lupus erythematosus. DI‐SCLE are associated with antihypertensives and antifungals, such as hydrochlorothiazide, diltiazem, and terbinafine.^1^ These drugs are hypothesized to induce a state of photosensitivity.^1^ Resolution of DI‐SCLE skin disease activity occurred in weeks following drug withdrawal, whereas antinuclear antibodies and anti‐Ro/SSA autoantibodies commonly remained positive for an extended period.^1^ Elimination of triggering medication and symptomatic management strategies—topical corticosteroid, systemic corticosteroid, and/or aminoquinoline antimalarial—are the main treatments for DI‐SCLE.^1^

Several cutaneous adverse events have been ascribed to conventional antituberculosis drugs. Ethambutol has been associated with lichenoid skin eruption, drug reaction with eosinophilia and systemic symptoms, Stevens–Johnson syndrome, and toxic epidermal necrolysis.^2^, ^3^ Previously published cases of DI‐SCLE were associated with isoniazid^4^ and rifampin.^5^ To the best of our knowledge, this is the first English article reporting ethambutol‐induced SCLE. In summary, we presented a case of ethambutol‐induced SCLE with characteristic clinical presentation, supportive histopathological features, and positive anti‐Ro antibodies. In this patient, the skin eruptions resolved after the interruption of the offending drug and the administration of systemic hydroxychloroquine and topical clobetasol.

## CONFLICT OF INTEREST

The authors declare no conflict of interest.
